# Biomedical Application, Patent Repository, Clinical Trial and Regulatory Updates on Hydrogel: An Extensive Review

**DOI:** 10.3390/gels7040207

**Published:** 2021-11-12

**Authors:** Sradhanjali Mohapatra, Mohd. Aamir Mirza, Ayah Rebhi Hilles, Foziyah Zakir, Andreia Castro Gomes, Mohammad Javed Ansari, Zeenat Iqbal, Syed Mahmood

**Affiliations:** 1 Department of Pharmaceutics, School of Pharmaceutics Education and Research (SPER), Jamia Hamdard, New Delhi 110062, India; sibanee@gmail.com (S.M.); aamir_pharma@yahoo.com (M.A.M.); 2 International Institute for Halal Research and Training (INHART), International Islamic University Malaysia, Kuala Lumpur 53100, Malaysia; ayah.hilles90@gmai.com; 3 Department of Pharmaceutics, School of Pharmaceutical Sciences, Delhi Pharmaceutical Sciences and Research University, New Delhi 110017, India; foziyahzakir@gmail.com; 4 Centre of Molecular and Environmental Biology (CBMA), Universidade do Minho, Campus de Gualtar, 4710-057 Braga, Portugal; agomes@bio.uminho.pt; 5 Institute of Science and Innovation for Bio-Sustainability (IB-S), Universidade do Minho, Campus de Gualtar, 4710-057 Braga, Portugal; 6 Department of Pharmaceutics, College of Pharmacy, Prince Sattam Bin Abdulaziz University, Alkharj 11942, Saudi Arabia; mj.ansari@psau.edu.sa; 7 Department of Pharmaceutical Technology, Faculty of Pharmacy, Universiti Malaya, Kuala Lumpur 50603, Malaysia; 8 Centre for Natural Products Research and Drug Discovery (CENAR), Universiti Malaya, Kuala Lumpur 50603, Malaysia

**Keywords:** hydrogel, biomaterial, patents, injectable, regulatory, drug delivery

## Abstract

Hydrogels are known for their leading role in biomaterial systems involving pharmaceuticals that fascinate material scientists to work on the wide variety of biomedical applications. The physical and mechanical properties of hydrogels, along with their biodegradability and biocompatibility characteristics, have made them an attractive and flexible tool with various applications such as imaging, diagnosis and treatment. The water-cherishing nature of hydrogels and their capacity to swell—contingent upon a few ecological signals or the simple presence of water—is alluring for drug conveyance applications. Currently, there are several problems relating to drug delivery, to which hydrogel may provide a possible solution. Hence, it is pertinent to collate updates on hydrogels pertaining to biomedical applications. The primary objective of this review article is to garner information regarding classification, properties, methods of preparations, and of the polymers used with particular emphasis on injectable hydrogels. This review also covers the regulatory and other commerce specific information. Further, it enlists several patents and clinical trials of hydrogels with related indications and offers a consolidated resource for all facets associated with the biomedical hydrogels.

## 1. Introduction

Hydrogels are polymeric matrices having a three-dimensional configuration that absorb water and undergo swelling but do not dissolve (short term) and facilitate the controlled drug-release into our body [1]. The high thermodynamic affinity of this class of materials towards the solvent is contributed towards its swelling property. They bear a physical resemblance to living tissues because of their significant water content and consistency. There is also a unique type of hydrogel, called as intelligent gels or smart gels. They can perceive the stimuli and respond by exhibiting changes in their physical or chemical behaviors and subsequent release of the entrapped drug. Hydrogels are widely explored as a tool for biomedical applications due to their ability to form crosslink under mild conditions coupled with high versatility, excellent biocompatibility, and permeability to oxygen, nutrient and tunable material’s properties.

Hydrogels can be delegated as a tool that meets the particular necessities to focus on the medication to the specific region and control its release. Properties of a hydrogel such as hydrolytic, enzymatic and ecological play a role to control the delivery of medication with the desired objectives such as extending their drug release profile, and expanding the choice of medicaments different drug delivery applications [2]. The hydrophilic functional groups attached to the polymeric backbone of hydrogels are responsible for their affinity to absorb water. Crosslinking between the network chains is answerable for its resistance to dissolution [3]. Both natural and synthetic materials can be used in the preparation of hydrogels. Synthetic hydrogels have replaced their natural counterparts from the last few decades due to their high water-absorbing capacity, long shelf life, high gel strength, and better stability profile in sharp fluctuating temperatures. Again, well-defined structures of synthetic polymers can be modified to yield tailor-able functionality and degradability [4].

**Figure 1:**
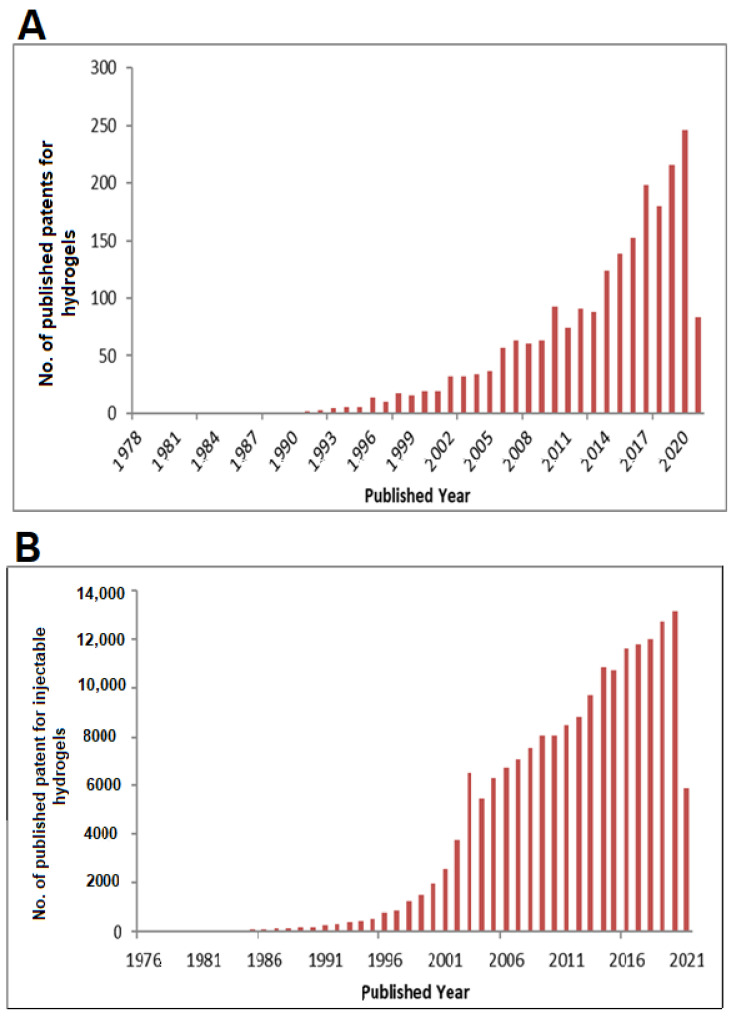
Histogram representing the published patents related with the hydrogels (**A**); and injectable hydrogels (**B**).

Hydrogel has a long history of evolution and has been reported upon with diverse classifications. The term hydrogel was initially coined in the year 1894 by Van Bemmelen [5]. The first-ever synthetic hydrogel poly(2-hydroxyethyl methacrylate) (pHEMA) was synthesized by scientists DuPont in 1936, and the first drug delivery hydrogel Cervidil^®^ for cervical ripening was launched in 1995 [5]. Since then, continuous research and development in hydrogel has led to a total of 329, 350 patents filed until today. However, the first generation of hydrogels aimed at developing artefact with high swelling and good mechanical properties with relatively simple rationale. It comprises chemical modifications of a monomer or polymer with an initiator by involving many cross-linking procedures. In comparison, the second generation of hydrogel can produce a response concerning specific stimuli, such as variations in temperature, pressure, light, magnetic fields, electrical fields, pH or concentration of specific molecules in solution. These specific stimuli can be exploited to trigger specific events. However, the third generation of hydrogels focusing on the study and development of stereo complexed materials (e.g., PEG-PLA interaction) [6,7] or formed by crosslinking due to other physical interactions [8,9]. Nowadays, research focuses on developing the so-called “smart hydrogels”, polymeric matrices having a broad spectrum of tunable properties. Nevertheless, there are several difficulties associated with utilizing hydrogels such as hydrophobicity of the active ingredients, which may create problems during medication conveyance during treatment. The water-cherishing polymeric center is most likely not an ideal site to hold incongruent hydrophobic medications. Other hydrogels have frail elasticity, which occasionally causes early arrival of the medication before landing at the objective site. In recent years, research on hydrogel has been continuously increased which can be evident from the Figure 1. Further, these statistics reveal a many fold increase in the number of patent publications related to the injectable hydrogel as compared to hydrogel from the last two decades.

Hydrogel controlled medication delivers the active constituent by utilizing framework gadgets that permit diffusion of medication and discharge through a cross-section (pores) that are loaded up with water. In the supply delivery framework, the hydrogel film is covered on a medication containing center, creating sections, cases and circles or having a high medication focus on the focal point of the framework to encourage a steady medication discharge rate. While the supply delivery framework produces time-free and consistent medication discharge using the macromolecular lattice. Lattice delivery is time-subordinate medication discharge in which the underlying delivery rate is relative to the square base of time instead of being steady. The drugs are dispersed in a polymer, and when they come into contact with water or bio-fluid they will begin to swell. During swelling, it will expand, which will ease the spread of the drug along with the relaxation of the polymer chain that supports time-independent and constant drug release kinetics. The active ingredient diffuses from the dispersed drug having a higher concentration within the hydrogel to its surrounding environment with lower concentration due to the concentration gradient. This method combines both the processes of diffusion and swelling for enabling drug release [10].

The following mathematical model can explain the process of swelling of a hydrogel matrix:

dc/dt = ΔD(t)Δc−Δ(cu)(1)

where, dc/dt = change in concentration with time, 

c = concentration of drug in the hydrogel

D(t) = diffusion coefficient

Δc = concentration gradient

u = swelling capacity of the hydrogel.

The diffusion process is governed by Fick’s law of diffusion which states that diffusion occurs from a region of high concentration to the low concentration:

dc/dt = χd2c/dx2(2)

where χ = diffusivity, 

t = time

x = dimension (in length)

**Figure 2:**
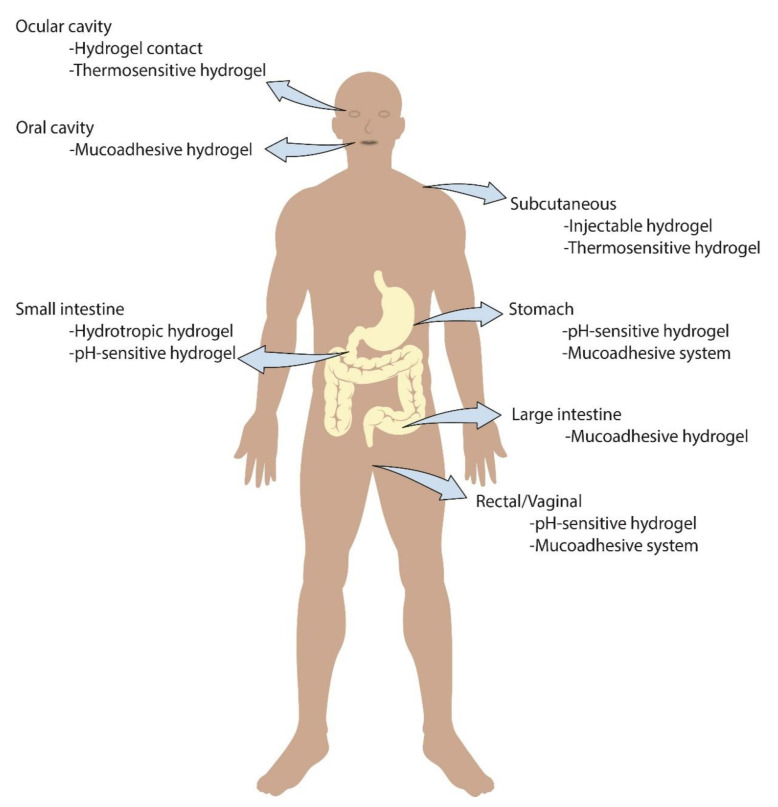
Applications of hydrogels in different human body parts.

Hydrogels significantly improve the remedial result of medication conveyance and have discovered gigantic clinical use. The worldly and three-dimensional conveyance of macromolecular medications have extraordinarily improved through hydrogel used for drug delivery [2]. Even though medication conveyance utilizing hydrogels has not been liberated from difficulties, consistent upgrades are being made to distinguish the hydrogel configuration most appropriate for explicit medication delivery purposes. However, hydrogels have recently drawn great attention for use in a diversified biomedical field for various applications such as the sustained release of active medicaments, cell therapeutics, cosmetics use, tissue regeneration and wound healing, etc. The following figure, Figure 2, illustrates the application of various hydrogels used in different body parts of humans. 

This review brings together different aspects of the hydrogel, such as classification, properties, preparation methods, the polymer used, and applications in the biomedical field. It specifically elaborates the injectable hydrogels enlisting their formulations for mitigating diseases. Further, it emphasizes on the regulatory aspects of commercial hydrogel highlighting the information related to certain ingredients. Additionally, it encompasses several patents, clinical trials and existing commercial hydrogel products with related indications. This review may act as a resource for the hydrogel system concerning the biomedical area covering all the essential aspects and may pave the way to conduct future research in this particular field.

## 2. Classification of Hydrogel-Based System

**Figure 3:**
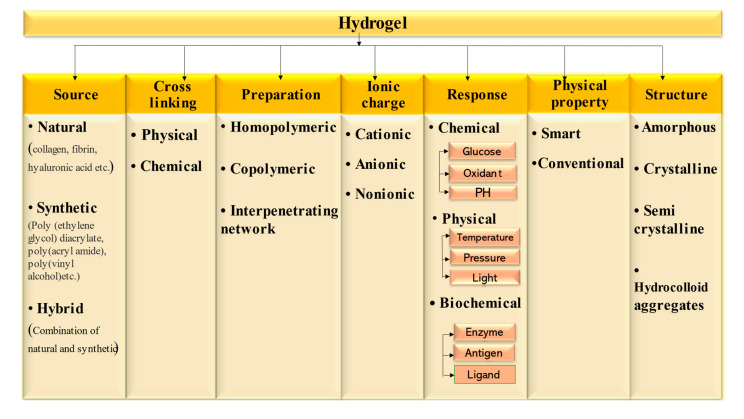
Classification of hydrogel-based systems.

There are several opinions found in the literature concerning the classification of hydrogel. They may be classified on the basis of the source from which they are obtained, physical properties, structures, crosslinking present, the ionic charge on bound groups, preparation methods and stimuli given to produce a specific response. Based on the above, the classification of the hydrogels are shown in Figure 3.

## 3. Polymers Used for Fabricating Hydrogel

Natural polymers are usually composed of components of proteins and extracellular matrix or derivatives of natural materials such as alginate, chitosan and skill fibers which makes them inherently bioactive, biodegradable, biocompatible, nontoxic and promote many cellular functions for different biomedical applications. The two drawbacks associated with natural hydrogels are that they have poor mechanical properties. Additionally, it is unclear about the correlation between the mechanical properties and polymerization or gelation conditions. Further, they offer difficulty in being manipulated as they have a high batch variation that may lead to poor reproducibility. In contrast, synthetic hydrogels are more reproducible, although their final structure can also depend on polymerization conditions, demanding rigorous control of the preparation protocol. So, it can be concluded that synthetic hydrogels offer more flexibility for altering chemical composition and mechanical properties than natural counterparts and are more prevalent. A few commonly used natural and synthetic polymers for hydrogels are enlisted in the following Table 1 [11,12].

## 4. Properties of Hydrogel

It is imperative to have a basic understanding of the gel properties so that a suitable gel delivery system can be designed. The interactions between the gel and the solute molecules can be better understood after studying the following properties.

### 4.1. Swelling

Hydrogels are crosslinked macromolecular polymeric networks that can swell in a liquid medium. The swelled polymer acts as a filter which allows partial diffusion of solute molecules. The polymer network is able to retain the solvent by forming a gel and will not dissolve if crosslinked. The presence of a hydrophilic functional group attached to the backbone and the difference in the osmotic pressure between the gel phase and the solvent phase is responsible for water absorption by the hydrogel. In hydrogel, the presence of water determines the overall permeation of nutrients in and out from the gel. Thermoresponsive hydrogels are one of the other categories of hydrogels having high biomedical interest. At room temperature these appear as fluid but convert into viscous gel as they get exposed to the body temperature, which lengthens their staying time, hence prolonging their release rate. They have the ability to undergo phase transition or swell/deswell at ambient alteration of temperature. Concentrate solution of poloxamer with water is one good example of thermoreversible gel widely used for tissue engineering applications nowadays [13,14]. Further, the rate and degree of swelling, controls the release patterns of drugs and solvents from hydrogel polymeric networks.

Researchers use several methods to determine the relative free and bound water contents with respect to total water content. It indicates the swelling property of the hydrogel. Some of the common techniques used for routine investigation of water content in hydrogels are: small molecular probes, DSC and proton NMR. Additionally, evaluation of swelling properties of the hydrogel serves as a measure for many of their properties such as mechanical properties, degree of crosslinking, rate of degradation and many more. Evaluation of the swelling and swollen state stability may help distinguish between crosslinked gels and the non-crosslinked original polymer [15,16].

### 4.2. Mechanical Properties

Generally, the mechanical property of hydrogel is linked to their water contents and cross linking density. The stiffness of the gel can be increased by increasing the degree of crosslinking or can be decreased by heating the material. An ideal hydrogel should be mechanically robust with rapid diffusion and response rate. The mechanical properties can be changed by a wide range of variables and causes, so it should be analyzed based on the aim of the study, types of material and the condition, etc. The mechanical properties of the hydrogel can be determined by texture profile analyzer or a rheometer by calculating young modulus, Poisson modulus, storage and loss moduli, etc. Currently, more efforts have been made to construct hydrogels with substantial mechanical performance. Double-network, topological, nanocomposite, macromolecular microsphere composite and supramolecular hydrogels are among the successful strategies for fabricating high-strength hydrogel. These are promising multifunctional materials having sufficient and robust mechanical properties that can be used successfully as tissue engineering scaffolds. The degree of stiffness of the hydrogel is determined based on its application area, i.e., where it has to be applied. For example, to seed osteoblast cells, a more rigid material is required than for culturing adipocytes [17]. However, materials characterization, tensile and compressive tests are basic methods for mechanical performance evaluation [18,19].

### 4.3. Porosity and Permeation

This is another important property that can simply indicate the presence of a void cavity inside the bulk. There may exist smaller pores within the network or may be formed in hydrogels during synthesis (by phase separation). It is beneficial to control the porosity for several applications, such as the tunable release of macromolecules, optimal cell migration in hydrogel-based scaffolds, etc. It has been found that porosity is a significant factor that influences the swelling and drug release behavior of the hydrogels [20]. The presence of a porous structure improves the drug release while non-porous hydrogels led to a very slow release. Additionally, the presence of pores can help in sustain release drugs for prolonged periods of time [21].

Porosity can be assessed by theoretical methods, such as liquid displacement method, Archimedes method, etc., with the use of optical and electronic microscopy. Some other methods such as gas pycnometer method, gas adsorption, capillary flow porosity have also been reported. One of the other important assays is X-ray microtomography [22]. Microscopy is another technique that can be used in assays involving hydrogels by which surface morphology and topography can be assessed. It involves the use of optical microscope, scanning electron microscope (SEM), transmission electron microscope (TEM), tunnelling microscope, atomic force microscopy (AFM) [23]. Additionally, thermoporometry helps to determine pore size based on melting or crystallization point of water molecules confined into the pores of hydrogels. It has advantages over other techniques, as it analyzes the sample in dried state [24,25,26]. It is based on analyzing the thermodynamic behavior of water relating to its interaction with polymers and provide a range of pore sizes in nano scale [27].

### 4.4. Crosslinking

Although crosslinking is not a basic property of hydrogels, it affects all the other material properties. It has some important characteristics, as it makes the hydrogel mechanically strong, and heat and erosion resistant. It may influence the rheological parameters, hydration and diffusion through skin [28]. The degree of crosslinking can be interconnected to every characteristic of a hydrogel, however the nature of crosslinking can vary a lot. The hydrogel’s network can be obtained in many different ways such as physical crosslinking (by complex coacervation or ionic interaction), chemical crosslinking via crosslinker or by radiation crosslinking. By regulating the degree of crosslinking, we can control the property of the material and can optimize it for numerous applications from the same original polymer [29,30]. However, there are several disadvantages such as relatively inflexibility in their processing properties as they are insoluble and infusible [31].

## 5. Method of Preparation

Usually, hydrogels are prepared from hydrophilic monomers, but sometimes hydrophobic monomers are also used to achieve certain desirable attributes. Synthetic polymers are hydrophobic and used to provide mechanical strength and durability to the hydrogels. The main components to fabricate hydrogels are monomer, cross linker and initiator, along with water that acts as diluent to regulate the heat of the reaction. Crosslinking reactions in hydrogel can occur via different methods, such as utilizing reaction and ionizing radiation to produce free radicals that recombine, creating cross-links, entanglements, electrostatics, and crystalline formation. 

**Figure 4:**
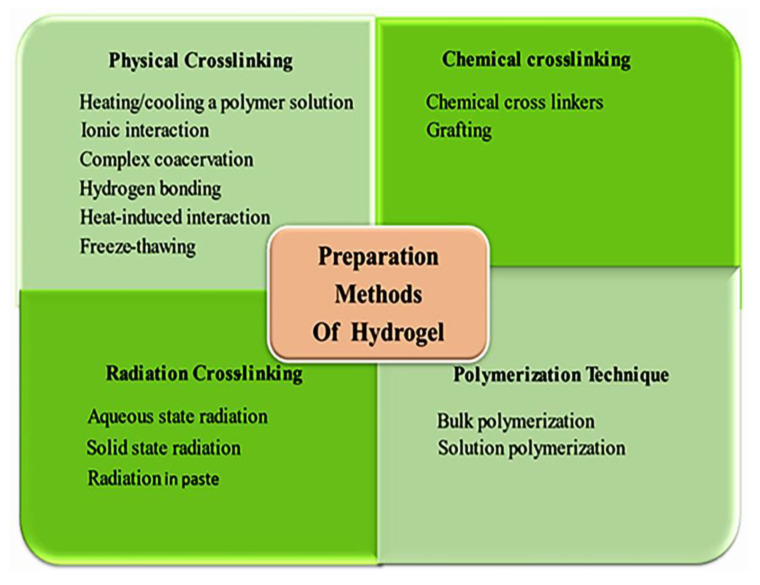
Different methods of preparation of hydrogel.

Hydrogels are derived from polar monomers and undergo crosslinking reactions by linking polymer chains to form networks. Such alterations can improve the mechanical properties and viscoelasticity for numerous biomedical applications in the pharmaceutical fields. The general methods to produce physical and chemical gels are summarized in Figure 4 [32].

## 6. Applications of Hydrogel

**Figure 5:**
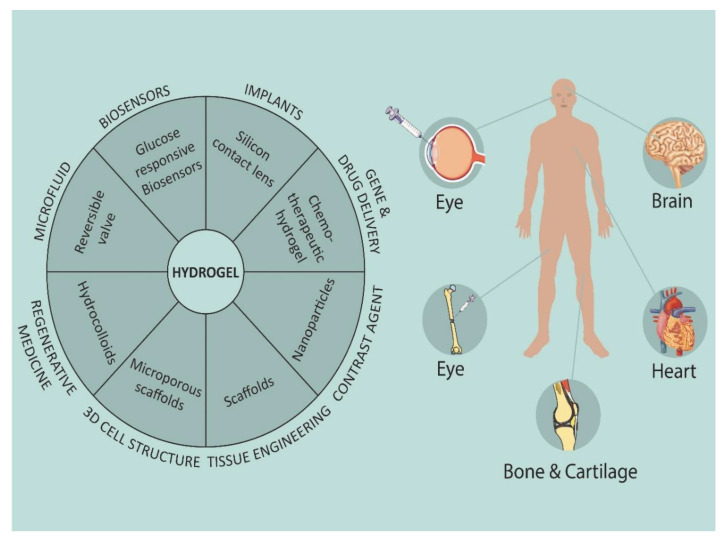
Possible biomedical applications of hydrogel.

Due to its versatility and flexibility, hydrogel possesses diversified applications such as biomedical, agriculture, sanitary diapers, dyes removal, heavy metal ions removal, biosensors, pH-sensors, and super-capacitors, etc. [33]. The following section includes biomedical applications of hydrogels in areas of cosmetic technology, wound healing, contact lenses, drug delivery, and tissue engineering, etc. [12,34]. Figure 5 mentions the biomedical applications of hydrogel. Further, this section includes a list of patents and clinical trials (Table 2 and Table 3) related to various hydrogel formulations, which give an idea about the current research conducted in this area.

### 6.1. Cosmetics Applications

Skin is the largest organ that acts as a physical and chemical barrier and protects the body. From the cosmetic point of view, skin is responsible for external appearance and is usually treated with cosmetic preparations. Skin condition depends on several factors, among which hydration of the skin is critical in maintaining its appearance and texture [35]. When the skin barrier is damaged, either due to climatic conditions and pollution or natural ageing (the genetic factors and photo-aging) it demands reconditioning. For this purpose, a range of moisturizers to re-establish the skin properties and barrier functions can be used. Stratum corneum (upper layer of the skin) can be rehydrated by three mechanisms such as by using humectants, occlusive and hydrophilic matrices. Nowadays, hydrogel is widely exploited in the cosmetics industries due to properties such as high water retention, biocompatibility, elasticity and softness [36]. These are mainly employed for skin hydration, wrinkles, pigmentation, cellulite and ageing, etc. Cosmetically these are mainly applied topically on skin, hair, and also used in oral care. Bioadhesive hydrogels are used for skincare purposes as they have advantages, such as long residence times on the site of application which ultimately reduces administration frequency. Acrylate-based hydrogel, due to its superabsorbent properties, is highly exploited to prepare hygiene products that can absorb fluids and keep moisture away from the skin, preventing diaper rash, promoting hygiene, and providing comfort.

### 6.2. Wound Dressings

Human skin has a unique potential for self-regeneration, owing to this, skin defects can heal spontaneously but if the defect is measuring more than a certain diameter, then it requires skin transplantation. Moreover, the wound healing process is impaired in some patients due to various conditions that lead to chronic wounds, which ultimately result in drastic conditions or even mortality [37]. Hydrogels are the most promising approach in wound healing amongst various wound dressings polymeric materials such as gauzes, hydrocolloids, gels, hydrogels, etc. Hydrogels act as an ideal wound dressing for the management of wounds as they can provide a moist milieu in the wound site, prevent infection, help in the removal of wound exudates and mimic the native skin microenvironment for tissue regeneration. In addition, hydrogels have unique features such as biocompatibility, softness and malleability which make them fit for the purpose. Polysaccharides based on hydrogel (cellulose, dextran, agarose) have greater water absorption capability, making them ideal candidates in wound healing [38]. However anti-bacterial and anti-inflammatory hydrogels have a good impact in wound healing [39,40,41].

### 6.3. Drug Delivery

Drug delivery can be defined as the process of administering a drug to a human or animal to achieve a desired therapeutic effect at an effective rate at the targeted site [42]. Delivery of the drug in a controlled manner at the particular target for an extended period is another important requirement of a good delivery system. Hydrogels, due to their three-dimensional release the drug in a controlled manner, especially for hydrophilic moieties. The porosity of the hydrogel further supports the sustained action of the drug [43].It also has a great potential for application via different routes. When the hydrogel is transplanted and injected or into an organism, it can maintain an embedded drug’s effective and controlled release into body fluids [44]. The hydrogel can improve the therapeutic effects of many lipophilic drugs that are restricted due to various problems, including poor solubility, poor dispersion, poor dissolution, low bioavailability, lack of uniformity, and lack of in vivo stability, etc. However, its fabrication is a difficult task and can be improved by the amalgamation of molecules having the ability to form inclusion complexes by incorporating the hydrophobic moieties [2,45]. Hydrogels can also be used as a carrier for biological macromolecular drugs, polysaccharide substances and genes, etc., without affecting the release kinetics. It follows different drug release models such as swelling controlled, diffusion-controlled and chemically controlled to release the medicaments in a controlled manner that act as release drives for the system [46].

Among diverse delivery systems injectable hydrogels are one of the extensively investigated scaffolds or therapeutic agents carriers in the area of disease treatment. 

#### Injectable Hydrogels for Disease Treatment

Due to numerous advantages of the hydrogel systems, they have been considered suitable scaffolds or active ingredients carriers [47]. However, it requires an invasive surgical procedure for implanting pre-formed hydrogels at a specific site in the body associated with pain and discomfort in the patients, that leads to decreased patient compliance and increased treatment costs; thus, limiting their clinical uses. Injectable hydrogels can overcome such disadvantages in biomedical applications with minimal invasiveness into target sites. These hydrogels can be exploited as a promising and efficacious material system for many applications in the medical field, such as treatment for cancers, inflammatory and infectious diseases; delivery of drugs, cells, and bioactive molecules and applications in the repair and regeneration of tissues such as skin, muscles, bone and cartilage [48]. Table 4 enlists various injectable hydrogels with biomedical applications.

Injectable hydrogels involve the sol–gel transition produced by cross-linking. For solutions of a polymer/monomer with therapeutic agents that have low viscosity when administrated to a desired site in the body, a hydrogel loaded with a therapeutic agent can be formed by crosslinking reaction that has a comparatively higher viscosity, called a gel state. The polymers in the hydrogel form a cross-link by chemical or physical interactions which are responsible for phase transition from solution to gel state. The sol-gel phase transition is sensitive to changes in pH, temperature, light, enzymes, ultrasounds, etc. [49,50,51]. Physical crosslinking occurs due to electrostatic ionic and hydrogen bond interactions, π-interactions, hydrophobic interactions, Van der Waals forces, etc. In contrast, chemical crosslinking occurs due to Schiff base reactions, Diels-Alder reactions, photo polymerizations, Michael additions, enzyme-mediations, etc.

Recently, the delivery of chemotherapeutics in the form of injectable hydrogels became a promising alternative for cancer/tumor management with biocompatibility, enhanced drug loading, prolonged and controlled drug release, with explicit stimuli sensitivity. Injectable hydrogel can be used as a flexible tool to reach some areas that cannot be easily touched by surgery. Furthermore, hydrogel can be used as a platform for tissue repair (tissue engineering [52,53,54]) and prevent postoperative tumor recurrence. Injectable biodegradable hydrogels that can form gels in situ have been widely utilized for biomedical applications. Additionally, these hydrogels can be biofunctionalized by targeting moieties that have an affinity for overexpressed and/or unique tumor cell markers for targeted drug delivery applications [55]. 

**Figure 6:**
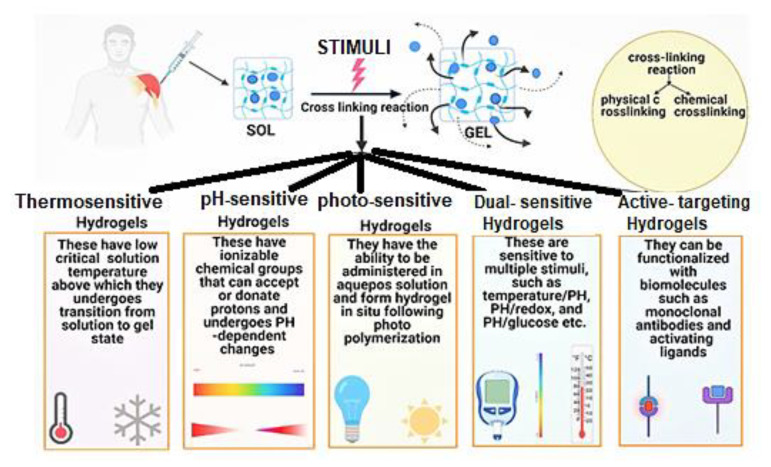
An overview of various aspects of injectable hydrogels.

Hydrogels have been used as a substitute for conventional cancer therapy, which is frequently associated with various factors such as unwanted toxicity to normal tissues, gigantic tissue loss, and unanticipated recurrence during or after the treatment. Localized drug delivery techniques (such as hydrogels [56], liposomes, nano/microparticles [57,58,59], micelles [8,60] offer localized sustained release of the chemotherapeutic. It results in increased efficacy of the treatment with minimal tissue toxicity by circumventing systemic circulation of the chemotherapeutic agents. They further facilitate high drug loading, improve solubility, and sustain drug release at the desire treatment location. Several systems have been explored for this purpose. The following Figure 6 depicts an overview of injectable hydrogel.

### 6.4. Tissue Engineering and Regenerative Medicine

Tissue engineering is a biomedical engineering discipline that refers to the practice of combining biologically active molecules, cells, and scaffolds into functional tissues. The main objective of tissue engineering is to accumulate biological substitutes that restore, repair, maintain, or improve injured tissues or whole organs. The scaffold resembles the extracellular matrix composed of porous structure responsible for supply of nutrients, cell growth and waste removal and thus aids tissue regeneration. In addition, the scaffolds should possess the desired quality attributes such as biocompatibility, biodegradability, mechanical strength and the ability to be sterilized. These characteristics are necessary for providing structural support to the cell and help in the process of cell growth and differentiation [92].

Regenerative medicine is a broad field where the body uses its own systems, occasionally with help foreign biological material to reconstruct cells and restructure tissues and organs. The terms “tissue engineering” and “regenerative medicine” can be used interchangeably, as the field focuses on cures instead of treatments for complex, often chronic diseases [34].

Although these areas currently play a relatively small role inpatient’s treatment due to lack of reproducibility and high cost but have a greater perspective in the field of drug development to screen different medications. Currently, these are used in the regeneration of cardiac tissues, cartilage, and bone [93]. Hydrogels represent a large class of materials that can function as tissue engineering to fabricate biocompatible and biodegradable cell scaffolds [94,95]. Properties of hydrogels such as structural similarities to natural extracellular matrix, delicacy and flexibility akin to soft tissue and ability to inject easily inside the body to form irregular non flowing gels are made it useful as a scaffold material in tissue engineering. The ability of the hydrogel to show electrical conductivity also plays an excellent role in tissue engineering [96]. Lyophilization, photolithography, microfluidic, micro molding, emulsification, solvent casting—leaching, gas foaming—leaching, and 3D printing are some approaches for the production of hydrogel scaffold [97].

### 6.5. Other Applications

#### 6.5.1. Hydrogel Machines

The unique properties of hydrogels, make them suitable candidate for fabricating different hydrogel machines for biomedical applications such as sensors, actuators, optics, coatings, electronics, etc. [98,99]. The basic requirement for this is the robustness of the hydrogels in terms of mechanical performance and functionality to ensure the stable operation of hydrogel machines. Recent innovations in the design of tough hydrogels [100,101], tough adhesion of hydrogels to other engineering materials, and advanced fabrication methods for hydrogels [102] have made hydrogels a promising material candidate for the next-generation machines [99].

#### 6.5.2. Biosensor

A sensor can be defined as a machine or part of it that detects and responds to signals in the environments [103]. Conventional sensors, (electronic sensors and electrochemical sensors) convert environmental inputs to electrical outputs based on semiconductors and/or metallic electrodes; whereas, hydrogel sensors are based on exclusive characteristics of hydrogels, such as high water content, stimuli-responsiveness, high compliance, and high permeability to a wide range of molecules. They can be classified into two types: (a) stimuli-responsive hydrogels (that can exhibit according to environmental inputs) [104,105]; and (b) passive hydrogels (as matrices to host responsive substances such as free ions, nanoparticles, biomolecules, and living cells, etc. that respond to environmental inputs). Hydrogel sensors are becoming practical tools for diverse applications including point-of-care detection, medical diagnostics, and environmental monitoring, etc. [105].

#### 6.5.3. Actuator

An actuator can be defined as a machine or a part of it that converts other forms of energy into mechanical energy to produce forces and motions. Conventional actuators adapted for mechanical systems usually contain metals and ceramics, and their actuation usually depends on relatively small deformations of the rigid materials [106]. Whereas hydrogel actuators provide mechanical motions commonly driven by relatively large deformations of the hydrogels [107]. Hydrogel actuators can be divided into three types such as (a) stimuli-responsive hydrogels driven by osmotic pressure change; (b) hydrogels matrices incorporating active elements (such as magnetic particles or free ions) in response to varying external fields (such as magnetic or electric fields); and (c) hydrogel structures with chambers.

#### 6.5.4. Coatings

In the human body, many tissues and organs are covered with hydrogel coatings, resulting in extremely slippery selectively permeable surfaces. For example, articular cartilage on bones provides a lubricated surface for smooth joint movement [108]. Similarly, when rigid machines such as orthopedic implants, neural probes, cardiac sleeves, glucose sensors, needles, catheters, ultrasound transducers, and electrodes for electroencephalogram, electromyogram, electrocardiogram, and transcutaneous electrical nerve stimulation come into direct contact with the human body, hydrogel coatings can potentially provide a biocompatible interface with minimal mechanical mismatch and foreign body responses. In order to coat hydrogels on engineering materials, including metals, ceramics, glass, and elastomers, adhesion needs to form between the hydrogels and engineering materials. According to different mechanisms of hydrogel adhesion, we classify hydrogel coatings into four types: (a) physical attached [109] (b) covalent anchored (c) interracially interpenetrated, and (d) mechanically interlocked [110] hydrogel coatings.

#### 6.5.5. Optics

Light and optical techniques have found particular importance in diagnosis, imaging, surgery, therapy, and many other biological types of research. Hydrogels is an ideal material for optic devices due to the above-discussed uniqueness, especially for those in close contact with biological organisms. There are two factors such as high transparency (low light absorption and scattering) and high refractive index (low bending loss) on which the effectiveness of hydrogel optics depends. According to their applications, there are four representative types of hydrogel optics developed so far, including (a) ophthalmic lenses [111], (b) smart windows and displays, (c) optical fibers [112], and (d) bioassay matrices [113].

Contact lenses are delicate ophthalmic tools that get direct contact with the eyes and are used to correct the vision, for cosmetic use imparting aesthetic effects and to deliver the active constituents for ophthalmic conditions [114]. Biocompatibility and permeability are two key properties to be considered during design besides comfort, permeable to ions for maintaining movement, continuous tear film for clear vision, non-irritable and resistance to tear film accumulation. Cornea needs oxygen for its proper functioning and therefore oxygen permeability is essential. Oxygen permeability is a very important property [115]. Principally there are two types of contact lenses, as rigid and soft contact lens.

Rigid contact lenses are typically made up of polymethyl methacrylate polymers which gives it properties such as wettability, elasticity and durability but lack of oxygen permeability. However a soft or hydrogel (poly(2-hydroxyethyl methacrylate) crosslinked with ethylene glycol dimethacrylate or silicone [116] having properties such as the ability to permeate water and oxygen with relatively high water content and good thermal and chemical stability [117]. They have the potential to assist in the treatment of various eye diseases, which is the reason why hydrogels are highly exploited as an important raw material for preparing contact lenses [118].

#### 6.5.6. Hydrogel Electronics

Due to their similarities to biological tissues and versatility in electrical, mechanical, and bifunctional engineering, hydrogels have recently attracted growing attention in bioelectronics to potentially provide a seamless interface between biology and electronics. Hydrogels are generally considered electrical insulators due to the absence of mobile charges or charge carriers. The electrical conductivity of hydrogels in the physiologically relevant conditions is similar to that of tissue media, and much inferior to common electronic conductors such as metals, limiting their applications in electronics [119]. To overcome such limitation and enable the possibility of hydrogel electronics for improved tissue-electrode interfaces, a few strategies have been developed to enhance the electrical conductivity of hydrogels, including (a) the addition of ionic salts in the hydrogels to achieve ionically conductive hydrogels (b) incorporation of electrically conductive micro-and nano-materials within hydrogel matrices to endow electronic conductivity [120,121], and (c) introduction of conducting polymers into hydrogels to enhance electronic conductivity [122,123].

The Table 5 depicts various commercially available hydrogel products with their respective indications which give an idea about the applicability of hydrogel in various field and pave the way for future research.

## 7. Regulatory Aspects of Hydrogel and Its Components

The diversified raw materials employed to develop hydrogel scaffolds make their regulatory arrangement and approval challenging. Unlike drugs which are broadly classified, hydrogels are classified under the “devices” category according to Section 201(g) of the FD&C Act. Furthermore, other than a few exceptions, most hydrogel-based products are required to undergo additional FDA review of a 510(k) pre-market notification submission for obtaining legal marketing rights in the United States, which takes many years to get regulatory approval. However, according to the new European regulation, hydrogels are considered as medical device class III, and it should be taken into consideration throughout the entire lifecycle of the hydrogel, starting from the material and machine qualification to scale-up. Table 6 enlists some regulatory information of hydrogel ingredients used for the manufacturing of hydrogels.

The commission regulation (EU) N0 722/2012 of 8 August 2012, concerning particular requirements as regards the requirements laid down in Council Directives 90/385/EEC and 93/42/EEC. These were concerning active implantable medical devices and medical devices manufactured utilizing tissues of animal origin, has adopted a regulation based on: The original requirements, the maintenance of a high level of safety and health protection against the risk of transmitting animal spongiform encephalopathies. The regulation also considers that class III’s active implantable medical devices and medical devices are subject to the conformity assessment procedures before being placed on the market or put into service, demanding the adoption of more detailed specifications relating to the risk analysis and management. The regulation establishes particular requirements concerning the placing on the market and/or putting into service of medical devices, including active implantable medical devices, manufactured utilizing animal tissue and their derivatives, originating from bovine, ovine and caprine species, deer, elk, mink and cats. In the case of collagen, gelatin and tallow used for the manufacturing of medical devices they shall meet at least the requirements as fit for human consumption. The regulation also establishes that the manufacturer of medical devices or his authorized representative shall carry out the risk analysis and risk management scheme before applying a conformity assessment. The member states shall verify that bodies have up-to-date knowledge of the medical devices to assess the conformity of those devices, and shall take all necessary steps to ensure that medical devices are placed on the market and/or put into service only if they comply with the current provisions and the particular requirements laid down in this regulation. Conformity assessment procedures for medical devices shall include the evaluation of compliance of the devices with the essential requirements into the current directives and the particular requirements laid down in this regulation. The manufacturer shall collect, evaluate and submit to the notified body information concerning changes regarding the animal tissue or derivatives used for the device or regarding the risk of the device. 

Two new European Health Products Regulations came into force on 26 May 2017. The first one, the Regulation (EU) 2017/745 of medical devices, which modifies Directive 2001/83/EC and subsequent derived regulations, repealing Council Directives 90/385/EEC and 93/42/EEC, had been applied from 26 May 2020. The second one, Regulation (EU) 2017/746 of in vitro medical devices, which repeals both Directive 98/78/EC and the commission 2010/227/EU decision, will apply from 26 May 2022. These new regulations represent an imperative change in the medical devices field and will necessitate stringent obligations to all market operators, which results in an increase in transparency and traceability guarantees of the product further leading to safety and reliability [155,156]. 

As hydrogels are considered a medical device class III by the new European regulation, this regulation must be considered not only in the scale-up process but also in the initial phases of the material’s and the machine’s invention. The regulation also considers that the active implantable medical devices and medical devices of class III are subject to the conformity assessment procedures before being placed on the market or put into service, demanding the adoption of more detailed specifications relating to the risk analysis and management. It is also considered for the elaboration of the regulation, the convenience of laying down additional provisions on the use of animal by-products not intended for human consumption, the adoption of several opinions on specified risk materials and on minimizing the risk of transmitting animal spongiform encephalopathy agents. The regulation considers that it is appropriate for the member states to verify that the notified bodies, designated to assess the conformity of those medical devices, have all the necessary expertise and up-to-date knowledge to perform this task. The period for scrutiny granted to the competent authorities of the member states concerning the notified bodies. The summary evaluation report should be shorter for medical devices manufactured using starting certified materials than in cases where uncertified materials are used. The regulation also bases its decisions on the convenience of the provision of an adequate transitional period allowing for active implantable medical devices already covered by an EC design-examination certificate or by an EC type-examination certificate to continue to be placed on the market and put into service, and that the measures provided for in this regulation are in accordance with the opinion of the Committee on Medical Device [157].

## 8. Conclusions

Hydrogels are a highly porous system and the polymers building them could be cross-connected to change degrees by altering their densities. Further, this porosity and the crosslinking ratio of hydrogel play a vital role in the release of medicaments. Applications of hydrogels are not simply restricted to focus medication conveyance they additionally discover applications in cleanliness items, wound dressings, contact focal points, tissue and machine designing. Further, it uses “smart” polymers capable of responding to various ecological signals permits administration of polymeric solution and undergo gelation under physiological conditions leading to in-situ hydrogels formation. However, preformed hydrogels are conventionally used for various applications as wound scaffolds; having the ability to release antimicrobial or anti-inflammatory drugs and growth factors from their structure by aiding the regeneration of the tissue. Additionally, hydrogels can be functionalized with a radiopaque that provides X-ray opacity and allows them to be used as biomedical implants for in vivo visualization and evaluation of the ability to prevent postoperative adhesions. Hydrogels are very versatile allowing varieties of the route for administrating drugs. In summary, hydrogels represent one of the most versatile technological platforms for pharmaceutical innovation. 

Ongoing improvements of hydrogels in the field of focused medication conveyance have been colossal. They are altered by focusing on ligands and different polymer types that present intriguing properties for drug transportation. Ophthalmic medication conveyance is a territory seeing the huge effect in treatment from hydrogels. From agreeable contact focal points to biodegradable medication conveyance the applications in eye care have been colossal. They are 90% water, give consistent drug discharge over days or months capable of conveying little particles to huge proteins, are completely invested in conveyance and stay noticeable during checking.

It is clear that there is a lot of potential in the field of hydrogels and number of setbacks which have to be overcome. Several investigators have shown significant results for improving the efficiency of hydrogel based drug delivery system. In tissue engineering fabrication technique based on in situ crosslinked hydrogel is much appreciated. Extensive studies have been conducted, and the number of patents published in this area of hydrogels has been increasing which is promising. Therefore, over the years, hydrogels became key players in the biomedical field in general and pharmaceutical research and development in particular, employing different invasive and minimally invasive administration routes. Future research in hydrogels will concentrate on the design of 3D structures with programmed bio functionality. There is a critical need to study major factors involved in the formation of hydrogels and to establish the different physicochemical criteria for the formation of reproducible, reversible 3D hydrogel networks with precisely defined structures and properties.

## Figures and Tables

**Table 1:** Polymers commonly used in various hydrogel formulations.

Natural Polymer	Synthetic Polymer	Hybrid Polymer
Hyaluronic acid	PEG-PLA-PEG	P (PEG-co-peptides)
Pectin	PEG-PLGA-PEG	P-(HPMA-g-peptide)
Alginic acid	PEG-PCL-PEG	P (PLGA-co-serine)
Carrageenan	PLA-PEG-PLA	Alginate-g-(PEO-PPO-PEO)
Chondroitin sulphate	PHB	HA-g-NIPAAm
Dextrin sulphate	P (PEG/PBO terephthalate)	Collagen-n-acryl ate
Chitosan	Polyurethane	Alginate-acryl ate
Polylysine	Polyimide	
Collagen (and gelatine)	Polyvinylpyrrolidone	
Carboxy methylchitin	Polyvinyl alcohol	
Fibrin and silk fibroin	Polyacrylate	
Dextrin	Polythene oxide	
Pullulan	Polymethacrylate	
Agarose Elastin Glycosaminoglycans Decellularized Hydrogels		

**Table 2:** List of Patent for various hydrogel formulation.

S. No.	Patent No./Country	Title	Disease/Problem	Details
1	US10799696B2 United States	Polymer formulations for nasolacrimal stimulation	Dry eye	The hydrogel formulation (prepared by a UV crosslinking process) permits electrical stimulation of the lacrimal gland, nasal or sinus tissue to ↑ production of tear and to treat dry eye
2	US20200085733A1 United States	Hypotonic hydrogel formulations for enhanced transport of active agents at mucosal surfaces	Administered into vagina or colorectum for diagnostic, prophylactic and therapeutic purpose	An aqueous polymeric hydrogel (poloxamers) used as a barrier by forming plug and/or used for the delivery to a mucosal/epithelial surface for therapeutic, preventive, diagnostic or nutraceutical purpose
3	US20200114010A1 United States	Non-injectable hydrogel formulations for smart release	The formulation contains anti-inflammatories, anti-infectives, or other therapeutic, prophylactic, or diagnostic agents that can be administered orally to produce desire action	A non-injectable formulation/formulation for instillation, with self-assembling hydrogels designed of gelators, in the form of capsules, tablets, oral suspensions, rectal or vaginal suppositories, enemas, and inserts
4	US20180023049A1 United States	Synthetic peptide hydrogel formulations for use as extracellular matrix	Cell culture experimentation	Synthetic peptide hydrogel solutions having a pH level of about 3.5/˂ and having a tonicity within an isotonic osmolality range
5	US20200360281A1 United States	A thermo-responsive hydrogel for intertumoral administration as a treatment in solid tumor cancers	Intra tumoral treatment of solid cancer	Injectable thermo-responsive hydrogel forming a chitosan and genipin interpenetrating scaffold by crosslinking can effectively incorporate chemotherapeutic drugs without any loss of thermo-responsiveness
6	WO2019067406A1 WIPO (PCT)	Biomimetic, moldable, self-assembled cellulose silica-based trimeric hydrogels and their use as viscosity modifying carriers in industrial applications	Use as low-cost and safe carriers and aqueous viscosity modifiers in various industrial and medical applications	A moldable, cellulose silica-based hydrogels which is fully scalable
7	US20190127726A1 United States	Delivering enzyme using an injectable hydrogel depot	To deliver enzymes	A delivery system for carrying an injectable enzyme hydrogel formulation consisting of an enzyme together with other components
8	WO2017152112A2 WIPO (PCT)	Hydrogel systems for skeletal interfacial tissue regeneration applied to epiphyseal growth plate repair	It can be applied through numerous different modalities depending on the nature of the physical injury	Biomaterials, systems, and methods for guiding regeneration of an epiphyseal growth plate or similar interfacial tissue structures
9	JP6293254B2 Japan	Silicone hydrogel lens with crosslinked hydrophilic coating	Contact lens	Coated silicone hydrogel contact lens containing a surface coating of silicone hydrogel and a non-silicone hydrogel which is a crosslinked polymer consisting of one or more cross-linkable materials and a crosslinked carboxyl-containing polymer material
10	CN105209016B China	Biocompatible hydrogel polymer matrices for cell delivery	A solid support that is beneficial for cell viability and functionality	Biocompatible hydrogel polymer matrices, bioabsorbable and releases cells at the target site, thus permitting controlled delivery
11	US20200299627A1 United States	Crosslinked hydrogel compositions for regulating states of encapsulated cancer cells	Method of regulating the state of cancer cells	A system composed of a crosslinked poly alkylene glycol based hydrogel, systems comprising a number of cancer cells in contact with the culture media and encapsulated inside the hydrogel, and the method of making and using the same
12	JP2020514500A Japan	Antibacterial polymer and antibacterial hydrogel	Antimicrobial polymers and antimicrobial hydrogels	An antimicrobial hydrogel containing a substituted C5-C15 alkyl; a polyethyleneimine-alkyl-polyethylene glycol methacrylate implant ratio ranging from 1:1:1 to 1:20:20 and a method of forming the same, providing a device having a surface coating the antimicrobial hydrogel as well
13	EP2801377B1 European Patent Office	Hydrogel comprising cells for local release of growth factors to mediate motor recovery after stroke	Hydrogel comprising cells that provide a sustained release of brain derived neurotrophic factor (BDNF) for improving recovery of a mammal after cerebral ischemia	Method of administering a therapeutically effective amount of BDNF to the infarct cavity in the mammalian brain for the treatment of cerebral ischemia
14	US20190282699A1 United States	Thiolated hyaluronan-based hydrogels crosslinked using oxidized glutathione	Hydrogel comprising the therapeutic agent, carboxymethylated hyaluronan and thiolated gelatin	Methods, compositions and kits linking to hyaluronan based matrices with oxidized glutathione as a crosslinking agent
15	WO2021019562A2 WIPO (PCT)	Bioengineered formulation, process for preparing and implementations thereof	Bioengineered formulation for corneal applications	A bioengineered formulation consisting of a modified collagen peptide and hyaluronic acid further, encompassing stem cells/exosomes or combinations therefrom
16	US10632070B2 United States	Hydrogel toxin-absorbing or binding nanoparticles	For ↓ or neutralizing the effect of a toxin, or for treating or preventing an infection by a microbe that produces a toxin, in a subject	Polymeric hydrogel formulation infused with a toxin-absorbing or binding nanoparticle
17	CN105979969B China	Topical compositions and methods of using the same	A topical pharmaceutical composition	A topical composition comprises a nitric oxide releasing active pharmaceutical ingredient mixed with a hydrophilic and a hydrophobic composition, in which the nitric oxide releasing active pharmaceutical ingredient encompasses a diazeniumdiolate (A nitric oxide releasing compound of a functional group)
18	JP6309458B2 Japan	Silicone hydrogel comprising N-vinylamide and hydroxyalkyl (meth) acrylate or (meth) acrylamide	Silicone hydrogel soft contact lenses provide improved oxygen permeability compared to soft lenses made from non-silicone materials	A silicone hydrogel comprising n-vinylamide and hydroxyalkyl (meth) acrylate/(meth) acrylamide
19	US10620456B2 United States	Increased stiffness center optic in soft contact lenses for astigmatism correction	Contact lenses for the correction of astigmatism	Contact lenses having a higher stiffness in the central optic zone for the correction of astigmatic refractive errors as well as possible higher order aberrations created by corneal geometry
20	JP6143269B2 Japan	Self-assembled composite ultra-small peptide polymer hydrogel	Topical agents for wound healing, as well as for delivering pharmaceuticals and other bioactive agents/components	A method for fabricating composite hydrogels, as implants/injectables that encourage tissue regeneration and as topical agents for wound healing to deliver pharmaceuticals and other bioactive agents components
21	US9937254B2 United States	Water-soluble supramolecular complexes	The complexes are useful in a variety of pharmaceutical and cosmetic products and may be combined with an effective amount of a cosmetic, medicament, or diagnostic in a solid dosage form	Water-soluble supramolecular complexes formed when combined with water, form a transparent thermo-reversible hydrogel/solution that may be repeatedly hydrated and dehydrated for sparely soluble and insoluble pharmaceutical agents, exhibits ↑ gelling efficiency, ↑ solubility and/or stability
22	EP2708224A1 European Patent Office	Biocompatible hydrogel polymer formulations for the controlled delivery of biomolecules	A biocompatible, bioabsorbable hydrogel polymer that releases the therapeutic agent at a target site, avoiding systemic exposure in a controlled delivery	The kits including at least one nucleophilic compound/monomer unit, minimum one electrophilic compound/monomer unit, and at least one drug. Further, the therapeutic agent such as a protein or other biomolecule is capable of gelling in vivo
23	JP2017527422A Japan	Composite materials for tissue repair	Composite materials and methods which restore lost soft tissue volume and promote soft tissue regeneration	A structural framework composite having a polymeric fiber component covalently bonded to a hydrogel material possessing ↑ properties
24	US20190343761A1 United States	Antibiotic formulations for lower back pain	Injectable, thermo gelling hydrogel formulations to relieve and/or treat low back pain	A thermosensitive hydrogel, consisting of an effective amount of an antibiotic, a radio-contrast agent, and at least 1 pharmaceutically acceptable excipient
25	US20170360912A1 United States	Chitosan-based hydrogel and applications thereof.	Chitosan-based hydrogel for medical and cosmetic treatments	flowable formulation and becomes a gel after a gelation time (depending on temperature) just immediately after preparation, containing chitosan, 0.4 M of sodium hydrogen carbonate (SHC), and a weak base different from the SHC
26	US10842743B2 United States	Modified hyaluronic acid hydrogels and proteins for the time-controlled release of biologic agents	Composition as a liquid capable of in situ formation of a hyaluronic acid-based hydrogel for treating a subject suffering from tumor(s)	Discloses the hyaluronic acid-based hydrogels, solutions for preparing same, and methods relating to this. It includes properties such as extended release, self-resorption of drug, and/or ↓ degradation, denaturation, and/or functional inactivation of active agents
27	US9211107B2 United States	Ruggedized ultrasound hydrogel insert	A ruggedized hydrogelproduct suitable for use in medical applications where sterile components are required	It contains a gel component, water for hydrating it, and minimum one free radical absorber component that has the capacity to absorb free radicals produced during the sterilization of the hydrogel through a high-energy sterilization procedure and can survive the effects of high-energy sterilization procedures, without substantial structural degradation
28	TWI558414B Taiwan	Thermosensitive injectable hydrogel for drug delivery	Heat-sensitive injectable hydrogels for drug delivery for delivering anti-cancer drugs	A heat-sensitive injectable hydrogel system based on hyaluronic acid and a copolymer of polyethylene oxide and poly oxypropylene, (having a gel formation temperature of 30 °C to 37 °C), providing an efficient drug delivery system that ↑ the therapeutic efficacy of the drug
29	JP6066237B2 Japan	Antibacterial ophthalmic contact lenses	Antibacterial ophthalmic devices made of hydrogel and epsilon polylysine (εPLL)	It comprises a hydrogel and at least 5μg εPLL bonded non-covalently to the hydrogel, the contact lens and the packaging solutions
30	EP3151872B1 European Patent Office	Wound dressing	A stimuli responsive wound dressing application against a wound site of a human or animal body	A wound dressing containing a lyophilized hyaluronic acid hydrogel and a number of implanted devices within hydrogel, each device includes chitosan and hypromellose where the formulation absorbs water and/or exudates and maintain a moist wound site which encourages angiogenesis and wound healing
31	EP3708167A1 European Patent Office	Immunomodulating treatments of body cavities	A combination medicaments for use in treatment of a cancer of an internal body cavity including urinary tract cancer, meant for local administration in a thermo-reversible hydrogel composition	A biocompatible hydrogel composition incorporating the combination of at least 2 immunomodulatory agents, where one or more of the therapeutic agents are embedded inside, and slowly released from it
32	WO2019221559A1 WIPO (PCT)	Microneedle adhesive patch based on hydrogel formulation	A microneedle patch that can be utilized for transdermal drug delivery to promote wound regeneration shows brilliant tissue adhesion, biocompatibility, and biodegradability	It comprises a 1st hydrogel layer with mussel adhesive protein and hyaluronic acid and 2nd hydrogel layer with silk fibroin, and a method for manufacturing it
33	WO2020036526A1 WIPO (PCT)	A biphasic hydrogel formulation and methods of production and use thereof	Creates an environment that relieves or encourages the healing process for the treatment of insect bites, erythema, pruritus, sunburn, acne, dry skin or callus	A hydrogel patch where a biphasic formulation is organized that encompassing a liquid layer externally and an elastic hydrogel in which the water formed on the surface of the elastic gel is physically cooling the skin by evaporation and give a 1st boost of the drug directly when placing on the skin
34	US20200246472A1 United States	Hydrogel-forming composition for controlled release	Drug delivery systems (injectable biogel)	Peptide hydrogelators capable of forming hydrogels as carriers of active ingredients/biological materials and act as sustained/controlled release systems
35	AU2015374022B2 Australia	Polyfunctional radical scavenger hydrogel formulation	Providing extended protection of the extracellular space within a wound site	The polyfunctional radical scavenger hydrogel formulation, A portion of the 1st radical scavenger included with the formulation and/or 2nd radical scavenger included within the formulation either in dissolved, suspended and/or bonded to a polymer of the hydrogel
36	US10471181B2 United States	Fiber-hydrogel composite surgical meshes for tissue repair	A surgical scaffold device for reducing foreign body response, managing tissue-materials interface, and improving the integration of the surgical mesh with the surrounding tissue of a subject	It disclose a composition and methods for a hydrogel/ nanofiber-hydrogel composite integrated with a surgical scaffold or mesh

**Table 3:** List of Clinical trials relating to various biomedical applications of hydrogel.

Type of Hydrogel	Disease	Formulation	Study Outcome	Status	Clinical Trial Identifier
Hydroxyethyl cellulose hydrogel	Knee pain by osteoarthritis	Injection	NA	On-going	NCT04061733
Polyacrylamide	Knee pain by osteoarthritis	Intra-articular injection	Clinical examination reported a transition from −7, meaning worse to 7, better on a scale of −1 to 7.	Completed	NCT03060421
Polyacrylamide hydrogel and hyaluronic acid	Knee pain by osteoarthritis	Intra-articular injection	NA	On-going	NCT02763956
Polyacrylonitrile hydrogel	Degenerative disc disease	Intra-discal	NA	On-going	NCT02763956
Hydroxyethylcellulose hydrogel	Knee pain by osteoarthritis	Intra-articular injection	NA	On-going	NCT04061733
Extracellular matrix hydrogel	Heart failure				
Alginate hydrogel	Heart failure	Intra-myocardial injection	Improved maximum oxygen uptake	Completed	NCT01311791
Renal cells gelatin hydrogel	Kidney disease	Injection	Improved levels of creatinine, proteinuria, GFR	Completed	NCT02525263
Renal cells gelatin hydrogel	Congenital chronic kidney disease	Injection	NA	On-going	NCT04115345
Human amniotic epithelial cells hydrogel	Asherman’s syndrome	Intra-uterine injection	NA	On-going	NCT03223454
Cardiac stem cells gelatin hydrogel	Ischemic cardiomyopathy	Intra-myocardial injection	Improved ventricular dysfunction	Completed	NCT00981006
Radiopaque Hydrogel	Pancreatic cancer	Injection	NA	On-going	NCT03307564
Biosentry Hydrogel	Pneumothorax risk after Lung biopsy procedures	Tract plug	NA	On-going	NCT02224924
TraceIT hydrogel	Oropharyngeal cancer	Injection	NA	On-going	NCT03713021
TraceIT hydrogel	Rectal cancer	Transperineal injection	NA	On-going	NCT03258541
SpaceOAR hydrogel (PEG)	Prevention of radiation exposure to rectum in radiation therapy	Injection	Reduced adverse effects and limited radiation exposure observed in subjects	Completed	NCT01538628
SpaceOAR hydrogel	Image Guided Intensity Modulated Radiotherapy for prostate cancer	Injection	Reduced rectal toxicity was observed following radiation therapy	Completed	NCT02212548
TracelT hydrogel	Bladder cancer radiation therapy	Injection	Helped in locating bladder tumor during imaging process	Completed	NCT03125226
VentriGel	Myocardial infarction/heart failure	Trans-endocardial injection	Parameters such as ejection fraction, end-diastolic volume and end-systolic volume were improved in myocardial infarction patients.	Completed	NCT02305602
Gut Guarding Gel (alginate with calcium lactate)	Endoscopic Submucosal Dissection	Sub-mucosal injection	It enhanced the mucosa formation and reduced bleeding/tissue injury following endoscopy	Completed	NCT03321396
Polyacrylamide hydrogel	urinary incontinence	Transurethral injection	The bladder retention volume was monitored and successful voiding was observed	Completed	NCT02776423
Polyacrylamide hydrogel and botox	urinary incontinence	Midurethral injection	Micturitions per day increased and relief from urinary incontinence observed	Completed	NCT02815046
Polyacrylamide hydrogel	Anal incontinence	Transanal injection	Reduced Wexner scores were observed after treatment	Completed	NCT02550899
OTX-TKI (polyethylene glycol hydrogel with tyrosine kinase inhibitor)	Age-related Macular Degeneration	Intravitreal injection	NA	On-going	NCT03630315

**Table 4:** List of various injectable hydrogels having biomedical applications.

Hydrogel	Active Ingredient	Type of Disease	In Vitro Cell Line	In Vivo Model	Conclusion	Reference
Thermosensitive chitosan-based	Disulfiram(DSF)	Cancer	Human HCC cell lines (SMMC-7721 cells)	-	A novel injectable sustained formulation for anticancer drugs aimed at the delivery of DSF for long-term cancer treatment	[61]
Dual thermo-and pH-sensitive injectable hydrogels of chitosan/(poly(N-isopropylacrylamide-co-itaconic acid)	Doxorubicin	Breast cancer	MCF-7 cells	-	Cytocompatible and exert no/negligible cytotoxicity on MCF-7 cells and has the potential for local therapy of breast cancer	[62]
pH-sensitive poly(lactic acid-*co*-glycolic acid)-*b*-poly(ethylene glycol)-*b*-poly(lactic acid-*co*-glycolic acid) (PLGA-PEG-PLGA) triblock copolymers	Herceptin	Breast cancer	-	SK-BR-3 tumor bearing mice	Great potential for preventing the relapse of HER2+ breast tumors after breast-conserving surgery with ↑ therapeutic efficacy, ↓ side effects and ↑ patient compliance	[63]
pH-sensitive injectable-polysaccharide-based self-healing hydrogels	Doxorubicin	Hepatocellular carcinoma	HepG2(release of drug from hydrogel) L929 cells (Cytotoxicity test of the hydrogel)	-	Self-healing property with high drug-loading ratio could prolong their lifetime during implantation and provide the benefit of nominally invasive surgery	[64]
Dual pH- and temperature-responsive physically crosslinked injectable hydrogel	Cancer	Oncolytic adenoviruses	-	Human xenograft tumor models	Exhibited ↑ and long-term antitumor therapeutic effects in tumor models and might have potential for long-term cancer treatment	[65]
Novel palladium nanosheet (Pd NS)-based chemo-photothermalhydrogel (Pd Gel)	Palladium and doxorubicin	Cancer	-	Mouse	A novel anticancer strategy that allows the release of doxorubicin more precisely, eliminate tumor more efficiently and inhibit tumor metastasis more persistently	[66]
ABA triblock copolymers of vitamin D-functionalized polycarbonate and poly(ethylene glycol), that is, VDm-PEG-VDm were synthesized and employed to form physically crosslinked injectable hydrogels	Bevacizumab; Avastin	Cancer		HCT116 xenograft mouse models	Injection of the hydrogel was effective to show antimetastatic activity as that of 4× weekly injections of Avastin thus ↓ the injection frequency and may ↑ patient compliance to treat metastatic cancer	[67]
pH-responsive injectable hydrogels made of a supramolecular cross-link network	doxorubicin	Cancer	L929 mouse fibroblasts	-	Showed biocompatibility, controlled release profiles and tunable properties which show a ↑ potential as a drug-releasing material for localized treatments	[68]
Triblock Copolymers of Vitamin E-Functionalized Polycarbonate and Poly(ethylene glycol)	Herceptin	Breast cancer	Human breast cancer cell lines (antitumor specificity and efficacy)	BT474 tumor-bearing mice- (biocompatibility and biodegradability)	↑ potential for use in subcutaneous and sustained delivery of antibodies to ↑ therapeutic efficacy and/or ↑ patient compliance as compared to intravenous and subcutaneous delivery of Herceptin in solution form	[69]
pH-responsive injectable hydrogels with mucosal adhesiveness based on chitosan-grafted-dihydrocaffeic acid and oxidized pullulan	Doxorubicin	Colon tumor	Colon tumor cells (HCT116 cells)	-	Showed good drug release, effectively killing colon tumor cells, ideal candidates for development of colon cancer drug delivery carriers /mucoadhesive drug delivery systems	[70]
Alginate hydrogel system	Angiogenesis with vascular endothelial growth factor (VEGF)	Cardiovascular diseases	Human microvascular dermal endothelial cells		Act as a new generation of therapeutic delivery vehicle by combining long-term in vivo therapeutic advantages with minimal invasion to treat cardiovascular diseases	[71]
Dual-responsive (pH and ROS) injectable hydrogels encapsulating drug-loaded micelles	Amikacin, andNaproxen	Wound healing		SD male rats	Possess good biocompatibility with efficient antibacterial and anti-inflammatory action, ↑ the healing process and promising to be applied topically against various microbial infections	[72]
Alginate–chitosan hydrogels	IgG model antibodies and Fab antibody fragments	Applications in drug delivery and regenerative medicine	-	-	Offers controlled delivery of antibodies and antibody fragments and will be promising formulation for several applications in drug delivery and regenerative medicine	[73]
Dopamine-based and polydopamine crosslinked injectable hydrogels	Dopamine and metronidazole	Parkinson’s disease	-	mouse L929 fibroblast cells	Can be used as long-term, localized, sustained release injectable system for dopamine as well as anti-inflammatory drugs to treat Parkinson	[74]
Covalently crosslinked composite hydrogel embedded with microspheres		Soft tissue engineering	-	-	Can be exploited as a potential opportunity to use this injectable composite gel scaffold in protein delivery and soft tissue engineering applications	[75]
Gelatin-hydroxyphenyl propionic acid (Gtn-HPA) and hyaluronic acid-tyramine (HA-Tyr)-based hydrogels	Human epidermal growth factor (hEGF)	Ophthalmic applications			Hydrodynamic model, giving a normalized diffusion and release of hEGF and provide the most suitable explanation for the measured solute diffusion coefficient	[76]
Porous alginate gels	Peptide antigen	Immunotherapies	-	Nonobese diabetic mouse model of type 1 diabetes	A noninflammatory biomaterial system can generate antigen-specific, that may enable the development of new therapies to treat transplant rejection/autoimmune diseases	[77]
Self-healing injectable micelle/hydrogel composites quaternized chitosan (QCS) solution and benzaldehyde-terminated poly(ethylene oxide)-*b*-poly(propylene oxide)-*b*-poly(ethylene oxide) (PEO_99_-*b*-PPO_65_-*b*-PEO_99_, Pluronic^®^ F127 (PF127)) (PF127-CHO) solution	Curcumin	Wound dressing for joints skin wound healing		Female Kunming mice	Self-healing antibacterial adhesive hydrogels with good mechanical property offer significant promise as dressing materials for joints skin wound healing	[78]
Alginate-gelatin injectable hydrogel	Oligochitosan coated cerium oxide nanoparticles	Age-related macular degeneration	Human retinal pigment epithlium-19 (ARPE-19) and umbilical endothelium	-	Biocompatible and have ↑ potential in protecting cells from angiogenesis, apoptosis, and production of proinflammatory cytokines with controlled drug release	[79]
Decellularized injectable cardiac and skeletal muscle extracellular matrix hydrogel	-	Potential scaffolds for tissue regeneration and/or repair for treating myocardial infarction, heart failure and peripheral artery disease	-	-	Tissue specific biomaterial therapies with minimal invasion	[80]
Polysaccharide-based hydrogels(N-carboxyethyl chitosan and oxidized sodium alginate)	Neural stem cells delivery	Neurological disorders	Neural stem cells	-	Neural stem cells transplantation and management of neurological diseases	[81]
Non-degradable dendritic polyglycerol sulfate (dPGS) hydrogel	Dendritic polyglycerol sulfate	Osteoarthritis	-	-	Formulation having good viscoelastic properties and has the benefit of being much less easily displaced from its injection site	[82]
Conductive anti-oxidant hydrogels (N-carboxyethyl chitosan and oxidized hyaluronic acid-graft-aniline tetramer	Amoxicillin	Wound dressing	C2C12 myoblast cells (Cytocompatibility) Escherichia coli and Staphylococcus aureus (Antibacterial activity)	Male Kunming mice	Have good antibacterial, biodegradation, electroactive and free radical scavenging property to efficiently prevent the wound infection and can be designed as an electroactive injectable hydrogel with promising applications	[83]
Injectable poly(ethylene glycol) (PEG)–gelatin hydrogel	Murine adipose-derived stem cells	Wound Healing and tissue regeneration	-	Murine wound healing model	Significantly ↑ cell retention, ↑ angiogenesis, and ↑ wound closure and can be used for regulating stem cell behaviors in 3D culture, delivering cells for wound healing and other tissue regeneration applications	[84]
Polyplex Micelle-Loaded Injectable Hydrogels	MicroRNA-29	Intervertebral disc degeneration(IDD)		Rabbits (therapeutic efficacy on fibrosis Inhibition) Sprague-Dawley rats (In vivo delivery analysis)	Successfully stop the expression of matrix metalloproteinases, prevent the fibrosis process and reverse IDD in animal models	[85]
Collagen–chitosan-based hydrogel	Thymosin β4, (a 43-amino acid peptide)	Myocardial Infarction	Monolayers of BHK-21		Stimulate angiogenesis and epicardial heart cell migration can be considered as a *carrier* of other negatively charged active biomolecules and thus shows numerous applications	[86]
Chitosan hydrogel	Human placenta-derived mesenchymal stem cell -derived exosomes	Hindlimb Ischemia	-	Murine model	Can ↑ the retention and stability of exosomes and further ↑ the therapeutic effects that may facilitate the development of easy and effective approaches for assessing and enhancing the therapeutic effects of stem cell-derived exosomes	[87]
Sustained release, thermosensitive polymeric [poly(lactic acid-co-glycolic acid)-poly(ethylene glycol)-poly(lactic acid-co-glycolic acid) (PLGA-PEG-PLGA)]hydrogel	Avastin^®^	Posterior segment disorders	-	Rat	A promising candidate for ocular drug delivery of Avastin^®^ through intravitreal injection	[88]
Catheter-injectable hydrogel utilizing a polymer–nanoparticle crosslinking mechanism	-	Various therapeutic applications		Wistar rats	Biocompatible, cell-signaling and can be differentially released with distinct elution profiles, allowing precise control over drug delivery	[89]
Self-healing hydrogel based on chondroitin sulfate multiple aldehyde and N-succinyl-chitosan	Cells encapsulated in the hydrogel	Cell carrier and in tissue engineering		Rat model	Shows biodegradability, produced ↓ inflammatory response and having potential application as a cell carrier and in tissue engineering.	[90]
Physiological temperature-responsive controllable NO-releasing redox injectable hydrogel	Nitric oxide(NO)	Cardiovascular diseases	-	Mice	Significantly ↑ the angiogenesis and new blood vessels formation by regulating the sustained release of NO and redox equilibrium in animal model. It has a ↑ potential in preventing and treating diseases	[91]

**Table 5:** Commercially available hydrogel product.

S.No.	Product	Product Manufactured by/Marketed by	Type of Hydrogel	Active Component	Indications	Reference
1	AquaDerm™	DermaRite	Hydrogel sheet	2-Acrylamido-2 methyl-1 propane sulfonic acid sodium, Propylene Glycol, Poly (ethylene glycol) dimethacrylate, 2-Hydroxy-2-methylpropiophenone with 38–55% water	Minor burns, pressure ulcers and radiation tissue damage	[124]
2	DermaSyn^TM^		Amorphous hydrogel		Acute/chronic partial and full thickness wounds/ulcers having minimal exudate	
3	DermaGauze™		Hydrogel impregnated gauze dressing	Acrylate polymer	Acute/chronic partial and full thickness wounds having minimal exudate and wounds with tunneling or sinus tracts	
4	DermaSyn/Ag™		Water-based antibacterial silver Wound gel	Silver	Venous ulcers, tissue trauma, pressure ulcers, diabetic ulcers, surgical incisions Thermal burns, etc.	
5	Intrasite^®^ GEL	Smith and Nephew	Hydrogel	Carboxymethyl cellulose and propylene glycol	Ease gentle, effective autolytic debridement to prepare the wound bed in all types of wounds	[125]
6	Suprasorb^®^ G	Lohmann and Rauscher Global	Hydrogel film	Water (70%), acrylic polymers based on a taurate derivative, polyethylene, phenoxyethanol, transparent polyethylene carrier film	Used for the management of the first and second degree burns, dry fractures, ulcer of the lower leg, pressure ulcer, etc.	[126]
7	Neoheal^®^	Kikgel	Hydrogel sheet	Water (90%), polyvinylopyrrolidone, polyethylene glycol and agar, crosslinked by a beam of electrons.	Burns, ulcerations, bedsores and all types of skin damages where humid medium is favourable	[127]
8	Woun’Dres^®^ Collagen Hydrogel	Coloplast	Collagen Hydrogel	Polymers such as carbomer and collagen	Dry wounds and eschar	[128]
9	Purilon^®^			Water, calcium alginate and sodium carboxymethyl cellulose	First and second degree burns, leg ulcers, pressure ulcers, non-infected diabetic foot ulcers	
10	Simpurity^®^	Safe n’Simple	Absorbent hydrogel sheets	water, polyethylene oxide, polyvinyl alcohol, acrylate, polyurethane	Wounds with minimal to no exudate, skin burns and dry scabs	[129]
11	SimpurityHydroGel^®^		Impregnated Gauze Wound Dressings		First and second degree burns, pressure sores and leg ulcers	
12	ProfiDerm^®^	Dr. Derm Professional	Hydrocollagen face gel	Sea collagen and hyaluronic acid	Nourishes, hydrates, soothes skin, helps to regenerate the skin of the face, ↑ the elasticity and tones the tissues	[130]
13	Advanced génifique light pearl hydrogel melting 360 eye mask	Lancome Paris	Hydrogel eye mask		↓ the appearance of undereye bags, puffy eyes, undereye circles and rejuvenate the eye area	[131]
14	Advanced génifique hydrogel melting sheet mask		Hydrogel sheet mask	Water, glycerine, polyacrylate-13, bifidus extract	Moisturized face skin, and make it radiant, smoother, shiny and healthy	[132]
15	Water bomb hydrogel mask	Moria	Hydrogel mask	Sodium polyacrylate, glycerine, cellulose gum, water	Restore hydration at a deeper level, soothesand rejuvenate the skin	[133]
16	EautraSil™	Miacare™	Silicone Hydrogel Contact Lens	Hyaluronic Acid and Sodium Alginate	Effectively prevent hypoxia-related complications (corneal neovascularization, redness, and corneal epithelium-aging)	[134]
17	Confidence		Silicone Hydrogel Contact Lens with Dot Matrix Colour Printing Technology	Hyaluronic Acid and Sodium Alginate	Long lasting comfort	[135]
18	Charcoal Hydrogel under eye mask	ELF cosmetics	Hydrogel mask	Charcoal Powder, Green Tea Extract, Lavender Extract	Under eye skin protection and rejuvenation	[136]
19	SEVEN RX^®^	Mark’ennovy	Hydrogel lens	Bioinspired silicone hydrogel lens	Short sight and long sight	[137,138]
20	Gentle 59	Mark’ennovy	Bio-inspired hydrogel lens		Short sight and long sight	[137]
21	MaxvueHiToric^®^	Maxvue vision	Silicone hydrogel	Hyaluronic Acid	Astigmatism	[137]
22	ACTIVHEAL® HYDROGEL	Advanced medical solution Ltd.	Amorphous gel	A primary wound dressing contains 85% water	Dry and sloughy wounds with zero to low exudate such as pressure ulcers, leg ulcers, cavity wounds, graft at donor sites, post op surgical wounds, lacerations and abrasions	[139]
23	Nu-Gel^®^ Hydrogel with Alginate	Systagenix wound management	Hydrogel with alginate	Alginate	Helps in management of chronic wounds through all stages of healing. Manage dry, encrusted and necrotic, sloughy, granulating andepithelialising wounds.	[140]
24	SUPPRELIN^®^ LA	Endo Pharmaceuticals, USA	Implant	Histrelin acetate	Treatment of children having central precocious puberty	[141]
25	Cervidil^®^	Ferring Pharmaceuticals, Inc.	Cervidil (dinoprostone) Vaginal Insert	crosslinked polyethylene oxide/urethane polymer, dinoprostone	Initiation and/or continuation of cervical maturement in pregnant women	[142]
26	SQZgel™	MacroMed	Controlled-release oral tablets	chitosan and polyethylene glycol	Hypertension	[143]
27	Mebiol^®^ Gel	Cosmo Bio co ltd	Thermoreversible hydrogel	poly(N-isopropylacrylamide) and poly(ethylene glycol)	High transparency for cell observation, stem cell culture, cell implantation, organ/tissue regeneration, drug delivery, and non-cell culture applications	[144]
28	Gelrin C™	Regentis Biomaterial Ltd.	Photo crosslinked hydrogel	polyethylene glycol and human fibrinogen protein	projected for the reparation of focal defects in cartilage and/or osteochondral defects	[145]
29	Mebiol^®^ Gel	Cosmo Bio	Thermoreversible Hydrogel	Poly (N- isopropylacrylamide) and POLY glycol ethylene	Cell implantation, organ and tissue regeneration, stem cell culture, drug delivery, and non-cell culture applications	[146]
30	HyStem^®^ Hydrogel	ESI BIO	Hyaluronic acid UV light-controlled system	hyaluronic acid	3D cell culture for tissue engineering purposes and 3D printing applications	[147]
31	Corning^®^ PuraMatrix™	Corning Incorporated Life sciences	Peptide hydrogel	-	3D cell culture used for stem cell proliferation, tumor cell migration and invasion, and in vivo analysis of tissue regeneration	[148]
32	Biogelx™	Bioglex Ltd.	Simple, short self-assembling peptides	-	Create an optimal environment for the culture of a variety of cell types, deliver synthetic yet biologically-relevant alternatives to animal-derived 3D matrices for example matrigel and collagen	[149]
33	SpaceOAR^®^	Boston scientific	Absorbable Injectable hydrogel	-	Imaging of cancerous cells and protecting healthy cells from radiation induced damage	[150]
34	Bulkamid^®^	Contura International	Soft injectable, transparent, hydrophilic gel	Synthetic polyacrylamide and water	Stress urinary incontinence	[151]
35	Symphony^®^	Cypre’s	Stimuli–responsive hydrogels as sensors	3D photolithographic instrument	Animal cell culture, 3D imaging, iPSCs, ESc cell lines, etc.	[152]
36	Valleylab™	Medtronic	Covalent anchored coatings	Chitosan-hyaluronic acid-based hydrogel catheter	Postoperative adhesion	[153]
37	Bolt Bis-Tris Plus Gels	Thermo Fisher	Bioassay matrices(gel)	Polyacrylamide gels	Western blot transfer and analysis	[154]
38	Tadpole™	Nervena^®^	Hydrogel electrode	Ionically conductive hydrogels	Use in Parotidectomy and otologic Surgery	

**Table 6:** Regulatory information about some hydrogel ingredients [158].

Common Name	USP-NF Name	Preferred Substance Name (USFDA)	EP Name	CAS No.	Maximum Potency per Unit Topical Dose(as per USFDA)	Relevant Physico-Chemical Properties	GRAS Listed
Alginate	Alginic acid, sodium alginate	Sodium alginate	Alginate, sodium	9005-38-3	0.25% *w*/*w*	Dissolves in water and forms a viscous solution	✓
Collagen	Gelatin	Type II Collagen	Collagen	9007-34-5	8% *w*/*w*		✓
Gelatin	Gelatin	Gelatin	Gelatin	9000-70-8	350 mg		✓
PEG 200	Polyethylene glycol 200	Polyethylene glycol 200	Polyethylene glycol 200	112,607	39% *w*/*w*	Viscosity 3.9–4.8 mPas at 98 °C	✓
PEG 1000	Polyethylene glycol 1000	Polyethylene glycol 1000	Polyethylene glycol 1000	25,322,683	0.5% *w*/*w*	Viscosity 16–19 mPas at 98 °C	
PEG 1600	Polyethylene glycol 1600	Polyethylene glycol 1600	Polyethylene glycol 1600	25,322,683	29.7% *w*/*w*	Viscosity 28–36 mPas at 98 °C	✓
PEG 300	Polyethylene glycol 300	Polyethylene glycol 300	Polyethylene glycol 300	25,322,683	57% *w*/*w*	Viscosity 5.4–6.4 mPas at 98 °C	✓
PEG 400	Polyethylene glycol 400	Polyethylene glycol 400	Polyethylene glycol 400	25,322,683	99% *w*/*v*	Viscosity 6.8–8 mPas at 98 °C	✓
Polyacrylic acid	Poly(acrylic acid)	Polyacrylic acid	Polyacrylate	9003-01-4	196 mg	Viscosity 50–200 mPas at 20 °C	✓
Polyvinyl alcohol	Polyvinyl alcohol	Polyvinyl alcohol	Polyvinyl alcohol	9002-89-5	140 mg		✓
Polyacrylamide	Polyacrylamide	Polyacrylamide	Polyacrylamide	9003-05-8	5% *w*/*w*		✓

USP NF—United States Pharmacopeia (USP) and the National Formulary (NF); USFDA—United States Food and Drug Administration; GRAS—Generally recognized as safe.
